# Surgical management of peripheral nerve sheath tumours in children, with special consideration of neurofibromatoses

**DOI:** 10.1007/s00381-020-04703-6

**Published:** 2020-06-06

**Authors:** Julian Zipfel, Meizer Al-Hariri, Isabel Gugel, Karin Haas-Lude, Alexander Grimm, Steven Warmann, Michael Krimmel, Victor-Felix Mautner, Marcos Tatagiba, Martin U. Schuhmann

**Affiliations:** 1 Division of Paediatric Neurosurgery, Department of Neurosurgery, University Hospital of Tübingen, Hoppe-Seyler-Str. 3, 72076 Tübingen, Germany; 2 Department of Neurosurgery, University Hospital Tübingen, Tübingen, Germany; 3 Centre for Neurofibromatosis at the Centre of Rare Diseases, University Hospital and University of Tübingen, Tübingen, Germany; 4 Department of Paediatric Neurology, University Children’s Hospital Tübingen, Tübingen, Germany; 5 Department of Neurology, University Hospital Tübingen, Tübingen, Germany; 6 Department of Paediatric Surgery, University Children’s Hospital Tübingen, Tübingen, Germany; 7 Department of Maxillofacial Surgery, University Hospital Tübingen, Tübingen, Germany; 8 Neurofibromatosis Centre Hamburg, Department of Neurology, University Medical Center Hamburg-Eppendorf, Hamburg, Germany

**Keywords:** Malignant peripheral nerve sheath tumour, Neurofibromatosis type 1, Perineurinomas

## Abstract

**Introduction:**

Peripheral nerve sheath tumours in children are a rare and heterogeneous group, consisting mostly of benign tumours as well as malignant neoplasms. Especially in the paediatric population, diagnostics and indication for therapy pose relevant challenges for neurosurgeons and paediatric neurologists alike. Most paediatric cases that need surgical intervention are associated to neurofibromatosis type 1 (NF1).

**Methods:**

We retrospectively reviewed all paediatric cases treated at the Department of Neurosurgery in Tübingen between 2006 and 2017 for peripheral nerve sheath tumours. We analysed clinical signs, symptoms, histology, association to an underlying phacomatosis and sensory/motor function.

**Results:**

Of the 82 identified patients, the majority had NF1 (76.8%). Nine children bore a sporadic tumour without underlying phacomatosis (11%), 8 had NF2 (9.8%) and 2 schwannomatosis (2.4%), A total of 168 surgical interventions were performed, and 206 tumours were removed. Indication for surgery was in most instances significant tumour growth (45.2%) followed by pain (33.9%). New deficits led to surgery in 12.5% of interventions; malignancy was suspected in 8.3%. Histopathology revealed mostly neurofibromas (82.5%), divided into cutaneous neurofibromas (10.7%), infiltrating plexiform neurofibromas (25.7%) and peripheral nerve-born neurofibromas (46.1%). 12.1% of tumours were schwannomas, 2.9% MPNST, 1.5% ganglioneuroma (*n* = 3) and 1 hybrid-neurofibroma and perineurinoma each. Leading symptoms, such as pain and motor and sensory deficits, improved after 125/166 interventions (74.4%), remained unchanged following 39 interventions (23.2%) and worsened in 4 occasions (2.4%).

**Conclusion:**

Surgery is safe and effective for (neurofibromatosis associated) peripheral nerve sheath tumours in the paediatric population; however, management needs a multidisciplinary setting. We propose early surgical resection in paediatric patients with peripheral nerve sheath tumours with significant growth, or pain, or motor deficit, or suspected malignancy.

## Introduction

Peripheral nerve sheath tumours are a heterogeneous group, consisting mostly of benign tumours (such as neurofibromas and schwannomas) as well as malignant neoplasms (malignant peripheral nerve sheath tumour, MPNST). They can be associated with significant neurological impairment, including sensorimotor deficits, pain and other disabilities [32]. Tumours that warrant surgical therapy in the paediatric age group are mostly associated with neurofibromatosis type 1 (NF1) and less often neurofibromatosis type 2 (NF2).

Benign nerve sheath tumours in NF1 can either grow diffusely as plexiforme neurofibromas (PNF) with local infiltration of surrounding tissue and organs, or as neurofibromas clearly related to a defined peripheral nerve, either as a solitary manifestation or as a circumscribed local manifestation in a diffusely changed “neurofibromatous” nerve. Strictly, cutaneous PNF can occur already in toddlers or young children with NF1 and differ distinctly from the typical NF1-associated adult cutaneous neurofibromas that mostly occur after puberty. Cutaneous PNF infiltrate the skin and the subcutaneous tissue diffusely and continue to grow mostly significantly during the first 2 decades of life [10].

Solitary benign peripheral nerve sheath tumours in children are rarely described, and most cases published were neurofibromas [7, 19]

Schwannomas occur mostly in patients with NF2, schwannomatosis or as solitary lesions [17]. Perineurinomas are tumours with perineural differentiation which may or may not be in association to a peripheral nerve [26]. Glomus tumours on the other hand are endocrine lesions arising from non-chromaffin cells of the parasympathetic system. There is an association with NF1.

NF1 is a neurocutaneous, autosomal-dominant genetic disease with a prevalence of about 1/3000 in the paediatric population [10]. Apart from the central nervous system, any peripheral nerve and the skin as well as other organs can be affected. The interindividual phenotype can vary immensely [13]. Peripheral nerve sheath tumours are significant factors in the morbidity of the pathology [20]. Plexiform neurofibromas are a specific manifestation typical for and confined to NF1, and 50% of patients are affected [10].

NF2 is as well an autosomal-dominant genetic pathology, defined by bilateral vestibular schwannomas. Its incidence is about 1/10 as compared with NF1 [4]. In children with NF2, extradural peripheral nerve schwannomas that need a surgical intervention occur much less frequently than in NF1 patients, and plexiform schwannomas or cutaneous schwannomas, although existent, are certainly rare.

Schwannomatosis is a third entity of genetic peripheral nerve sheath tumour disposition. It is characterised by multiple peripheral schwannomas without the presence of bilateral vestibular schwannomas or other intradural NF2 manifestations like meningeomas or ependymomas [3, 29]. It can be associated to SMARCB1 or LZTR1 mutations and exists in a sporadic and a familial pattern. It however mostly affects adults, and an age < 30 years is considered an exclusion criterion for the sporadic form [9].

Malignant peripheral nerve sheath tumours (MPNST) most often arise from previously benign peripheral nerve neurofibromas or arise in extraneural soft tissue like muscles. In case of NF1, they mostly arise within plexiform neurofibromas but not from the purely cutaneous form [23]. MPNST show a developmental plasticity, and in adults, they partially arise in the post-radiation setting [26]. Predilection regions include proximal extremities, torso and cervical region. Clinical manifestations include painful and rapidly growing tumours with neurological deficits. Prevalence is at about 0.001% in the general population as compared with 5–10% in NF1 [5, 11, 25, 30]. Diagnostic imaging to differentiate between benign and malignant nerve sheath tumours is challenging and includes PET-MR/CT as well as MR-spectroscopy [1, 12, 28, 31].

The mere existence of a benign-looking asymptomatic, non- or slowly growing non-cutaneous peripheral nerve tumour, especially in the context of an underlying NF1/NF2 or schwannomatosis—a description that covers most peripheral nerve sheath tumours in children with the above-mentioned diseases—is not an indication for surgery.

Surgical resection of peripheral nerve tumours in the paediatric population is usually indicated in one of the following situations:

- Local painful tumour independent of size or pain radiating in the skin distribution of the affected nerve
- Increasing motor or sensory deficit in the distribution of the affected nerve
- Mass effect of a circumscribed tumour leading to impairment of movement or dressing with normal clothing or shoes
- Cutaneous PNF with documented growth
- Fast growth of any circumscribed tumour within the last or change of imaging characteristics indicating the possibility of hypercellar neurofibroma or MPNST in case of NF1
- Increasing infiltration of adjacent tissue of a PNF that either leads to mass effect, organ/ muscle dysfunction, bone destruction or impossibility of resection at a later time point due to critical infiltration
- Tumour growth that results in cosmetic disfigurement

In this study, we analyse the symptomatology and results of surgery for peripheral nerve sheath tumours in children and adolescents treated between 2006 and 2017. Especially, pre- and postoperative motor and sensory functions as well as effect on pain were the focus of the outcome analysis.

## Methods

We performed a retrospective analysis of all operated cases in our institution between 2006 and 2017 using database search after approval of the local ethics committee (Nr.: 026/2018BO2).

A total of 82 paediatric patients were identified who underwent 168 surgical interventions in the analysed period. Tumours operated in different areas of the body were counted as separate interventions, since each surgical site had its specific characteristics and risks, and sometimes different types of tumours were operated (e.g. a cutaneous PNF at the trunk and a solitary neurofibroma of the arm).

We analysed clinical signs and symptoms; histopathology; association with NF1, NF2 or schwannomatosis; and neurological deficits like sensory and motor dysfunction. We used the MRC scale grading system for motor function. Accordingly, sensory function was graded from 0 to 5 (no sensibility, severe hypaesthesia, moderate hypaesthesia, mild hypaesthesia, normal sensibility).

Pain was rated on an increasing pain scale from 0 to 5 (no, minimal, mild, moderate, severe pain, respectively).

Statistics were performed using SPSS Statistics 25 (IBM, NY, USA). Continuous data were presented as mean (± SD), whereas categorical data were shown as count with percentages in parentheses (*n*, %). Continuous variables were tested for equality of variances by Levene’s test. Normally distributed parametric variables with equal variances were compared using the unpaired or paired *t* test and ANOVA. Post hoc analysis was performed including Bonferroni correction. *P* values < 0.05 were regarded as significant. Descriptive data is provided including standard deviation.

## Results

A total of 82 patients were investigated. 57.3% of patients were female (*n* = 47) and 42.7% male (*n* = 35). Two hundred six tumours were removed in 168 surgical interventions during the observation period.

### Incidence of phacomatosis

The majority of patients had NF1 (*n* = 63, 76.8%), 9 children had a sporadic peripheral nerve sheath tumour (11%), 8 children had NF2 (9.8%) and 2 had schwannomatosis (2.4%)

Diagnosis of NF1, NF2 or schwannomatosis was established by fulfilling the necessary diagnostic criteria according to guidelines in 57/73 patients (76%); in the remaining 16 (19.5%), genetic testing confirmed the diagnosis in addition.

### Indication for surgery

For most interventions, the reason for surgery was significant tumour growth (*n* = 76, 45.2%) followed by pain (*n* = 57, 33.9%). Local symptoms and compression effects of vascular or neural structures existed prior to 21 interventions (12.5%), and malignancy was suspected in 14 interventions (8.3%).

In most operations, one single nerve sheath tumour or tumour conglomerate (in case of PNF) was resected (*n* = 148, 88.1%). Ten patients (6%) had 2 tumour interventions at one surgery, 5 (3%) had 3 interventions, 2 patients (1.2%) had 4 and in 3 patients (1.8%) had 5 interventions, leading to a final count of 168 interventions with 206 removed distinct tumour manifestations.

Ten cases were operated for a recurring tumour at the same site (6%). Complete tumour resection was possible in 130/168 occasions (77.4%).

### Histopathology

The histopathologic workup of 206 tumours revealed mostly neurofibromas (*n* = 170 (82.5%). This group could be divided into cutaneous neurofibromas (*n* = 22, 10.7%), plexiform neurofibromas (*n* = 53, 25.7%) or peripheral nerve neurofibromas (*n* = 95, 46.1%).

**Fig. 1:**
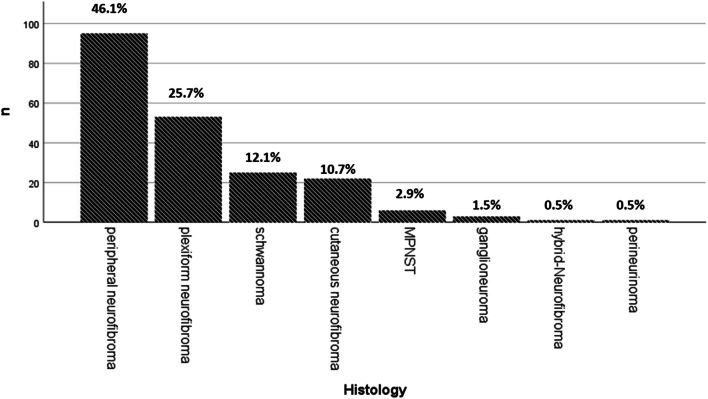
Overview of histological classification of resected tumours *n* = 206

There were 25 schwannomas (12.1%) and 6 MPNST (2.9%). Furthermore, we encountered three ganglioneuromas (1.5%), and one hybrid-neurofibroma (0.5%) and perineurinoma (0.5%), respectively. An overview is provided in Table 1 as well as in Fig. 1.

**Table 1:** Overview on 82 patients, in whom 168 interventions were performed to remove 206 tumours

	**Patients**	***n*** **(82)**	**%**
Syndrome	NF1	63	76.8
	NF2	8	9.8
	Schwannomatosis	2	2.4
	Sporadic	9	11.0
Sex	Female	47	57.3
	Male	35	42.7
	**Interventions**	***n*** **(168)**	**%**
Indication for surgery	Tumour growth	76	45.2
	Pain	57	33.9
	Local complications	21	12.5
	Suspicion of malignancy	14	8.3

In the 9 patients with solitary peripheral nerve tumours, we operated on 3 schwannomas, 2 ganglioneuromas, 2 MPNST, 1 peripheral neurofibroma and 1 perineurinoma.

All NF1 patients had neurofibromas, all NF2 and schwannomatosis patients had schwannomas. The hybrid-neurofibroma was found in a NF2 patient, the third ganglioneuroma in a child with NF1.

### Localization

**Fig. 2:**
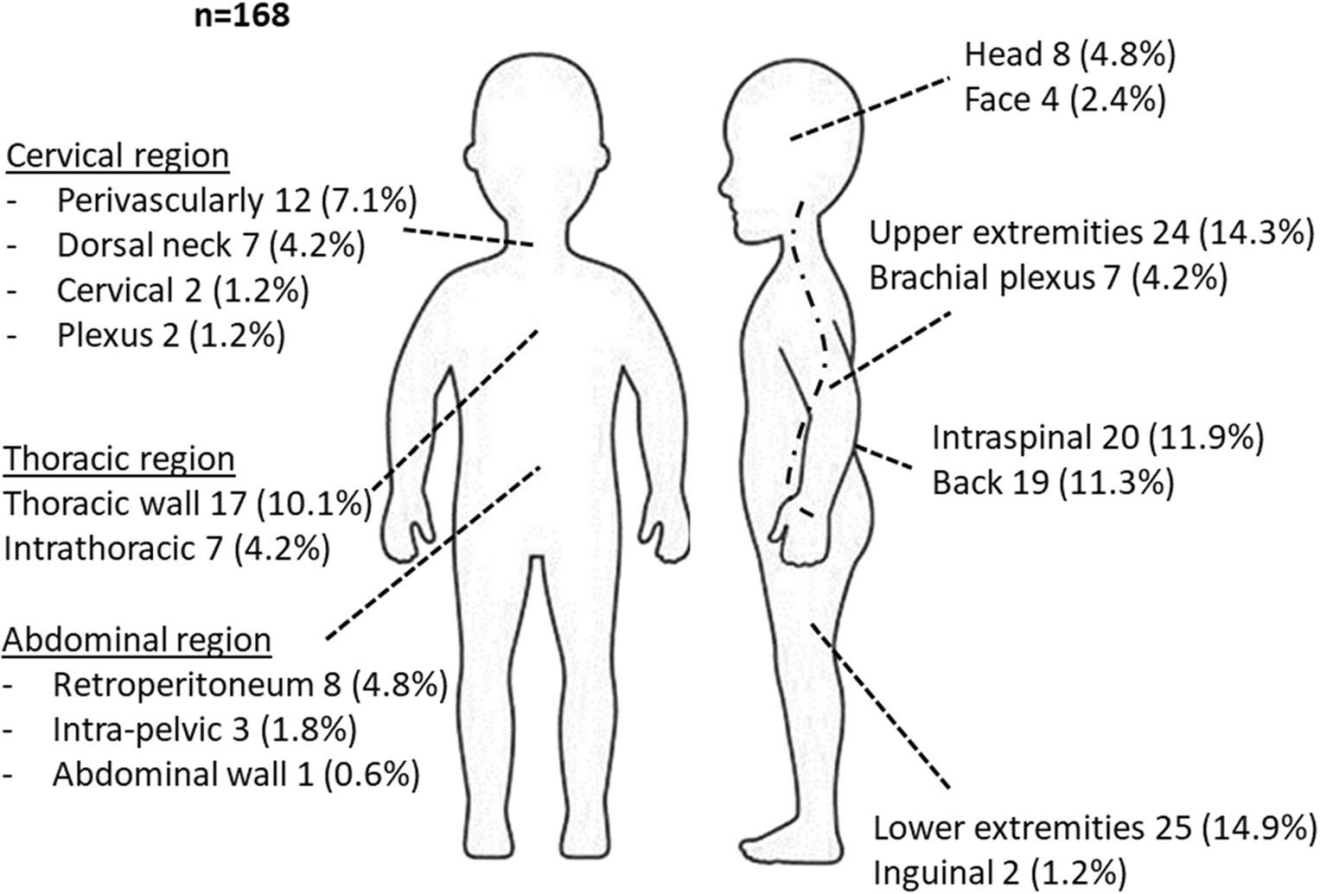
Overview of localization of surgical tumour resections *n* = 168

The anatomical localization of the 168 interventions is shown in Fig. 2.

### Preoperative symptoms and neurological status

Preoperative neurological evaluation showed in most cases good motor function. Grading was available for 166 interventions performed via the MRC scale. For 147/166 interventions, patients had full function of the corresponding nerves (5/5, 87.5%); in 10 interventions, minor paresis of the affected nerve (4/5, 6.0%) and, in 4, major paresis allowing for movement against gravity (3/5, 2.4%) were present. In 2 interventions (1.2%), movement was only possible with gravity eliminated (2/5), and in 3 (1.8%), only visible muscle contraction without limb movement was observed (1/5).

The observation for sensory function was similar. Prior to 146/166 interventions, sensory function was unimpaired (5/5, 86.9%); in 6, it was mildly impaired (4/5, 3.6%), and in 2, moderate hypaesthesia was reported (3/5, 1.2%). In 2 interventions, major sensory impairment was observed (2/5, 1.2%), and in 10, anaesthesia was present (1/5, 6%).

Pain was a major indication for surgery, being reported prior to 44 interventions (26.2%) as severe pain, for 39 (23.2%) as moderate pain and for 13 as mild pain (7.7%). Pain was not present prior to 72/166 interventions (42.9%).

### Postoperative outcome

Leading symptoms, such as pain and motor and sensory deficits, improved after 125/166 interventions (74.4%), remained unchanged following 39 interventions (23.2%) and worsened in 4 occasions (2.4%).

At last follow-up (mean 3.3 years), symptoms were still improved after 112/166 interventions (66.7%), unchanged after 45 (26.8%) and worsened after 7 (4.2%).

#### Motor function

Full motor function (5/5) was observed after 144/166 interventions (85.7%). A minor impairment (4/5) was present in 13 cases (7.7%). Four patients (2.4%) were only able to move their limb against gravity (3/5) and 1 patient had severe impairment (2/5, 0.6%), and in 4 cases, motor dysfunction was nearly complete (1/5, 2.4%).

#### Sensory loss

Sensory function was unimpaired postoperatively in 139/166 cases (5/5, 82.7%). In 13 cases (7.7%), mild sensory impairment was observed (4/5), 3 cases had a moderate impairment (3/5, 1.8%) and 11 cases had a major impairment (1/5, 6.5%).

#### Pain

Following 136/166 interventions, no pain was reported (81.0%). After 3 interventions, severe pain persisted (1.8%). Two cases reported moderate pain (1.2%), and 25 mild pain (14.9%) after surgery.

### Outcome according to tumour entity

When comparing the major groups of schwannomas, peripheral nerve neurofibromas and PNF, we can see that schwannomas present with significantly more pain than the other groups preoperatively, but not postoperatively. PNF have a significantly better preoperative motor status than the other groups.

In 21 interventions for schwannomas, preoperative motor function was unimpaired in all but one case (4.5%, MRC 3/5). Postoperatively, all patients had full motor function. Sensory function improved following 6 interventions (27.3%), was unchanged in 13 (59.1%) and worsened in 2 cases (9%).

Schwannomas showed significantly more pain than all other pathologies.

In cutaneous PNF, as expected, no pre- or postoperative motor impairments were found. All lesions were with intact skin sensation preoperatively. Postoperative sensory deficits were limited to the operative field.

In deeply seated PNF, postoperatively, no motor impairment was found, but in two patients, a significant worsening of sensory function occurred (5%).

Regarding peripheral nerve neurofibromas, 3 cases (4.1%) had a major preoperative motor deficit (MRC 1/5). One did not improve and two patients recovered significantly (up to MRC 4/5) after surgery. For three interventions (4.1%), moderate motor impairment was found preoperatively (MRC 3/5) without a significant postoperative change. The same was true for nine interventions (12.2%) with minor motor deficit (MRC 4/5). Of the 59 interventions (79.6 %) with preoperative full motor function, one patient (1.4%) experienced deterioration postoperatively.

Sensory function improved in 2 cases (2.7%), was unchanged in 64 (86.5%) and worsened in 8 cases (10.8%).

One of six patients with MPNST had a minor preoperative motor impairment that persisted postoperatively (MRC 4/5). The other 5 MPNST patients had full motor function preoperatively (MRC 5/5). In three postoperative motor function remained unchanged (MRC 5/5), one patient had a minor impairment (MRC 4/5) and one patient completely lost motor function (MRC 0/5). In this case, the L3 root had to be sacrificed due to malignant tumour invasion. Sensory function was unchanged in 5 and worsened in 1 case.

These 6 patients had significantly more preoperative sensory impairment than those with benign tumours (3.0 ± 2.2 vs 4.9 ± 0.6, *p* < 0.001). Postoperatively, this was unchanged (2.7 ± 2.0 vs 5.0, *p* < 0.001).

Preoperatively, but not after surgery, pain was significantly higher in MPNST patients (2.5 ± 0.5 vs 1.3 ± 1.3, *p* = 0.021).

Both MPNST and schwannomas showed a significantly lower preoperative mean motor function than NFib. Postoperatively, schwannomas showed a distinctly better motor function (4.5 ± 0.92 vs 4.6 ± 0.94, *p* = 0.029); however, it was still lower than in NFib.

The single patient with perineurinoma had severe preoperative motor and sensory impairment (2/5) which persisted postoperatively.

Of the three patients with ganglioneuroma, two had full pre- and postoperative motor function (MRC 5/5) and one experienced significant improvement (preoperative 2/5–postoperative 3/5). Sensory function did not change significantly

### Association of outcome to resection status

Preoperative mean pain level was significantly higher in patients in whom complete resection was not possible (1.8 ± 1.3 vs 1.2 ± 1.2, *p* = 0.010) as well as in patients with recurrent tumours (1.25 ± 1.25 vs. 2.4 ± 1.0, *p* = 0.005). The latter was also true postoperatively (0.2 ± 0.5 vs 0.8 ± 0.6, *p* = 0.001). Complete resection was possible in only 40% of recurrent tumours as compared with 80% of primary tumours (*p* = 0.003).

Additional surgical complication was rare. After two interventions (1.2%), local haemorrhage in the area of surgery occurred without the need for surgical intervention. One patient complained of a seroma, which spontaneously resolved after several days.

## Discussion

The literature on peripheral nerve tumour surgery in children and adolescents is scarce. Recently, a cohort of just seven paediatric patients with eight peripheral nerve tumours has been published [16]. Furthermore, purely larger paediatric series do not exist; in mixed series, paediatric cases are not evaluated separately [6–9, 18, 21, 27]. Due to the rare nature of peripheral nerve sheath tumours in children and its association with rare genetic pathologies like neurofibromatosis, only limited data exists. To the best of our knowledge, this retrospective analysis represents the largest series on peripheral nerve tumours in children so far. We are aiming at providing an overview of the underlying pathologies, treatment strategies and surgical outcomes.

### Phacomatoses as underlying disease

With regard to neurofibromatosis, the correct interpretation of the clinical significance of a certain pathology like neurofibroma or schwannoma is key to further management and diagnosis. The existence of a peripheral nerve tumour together with the other diagnostic criteria of schwannomatosis [24], NF1 [2] or NF2 [22] can pave the road to final diagnosis. After removal of a neurofibroma, a NF1 needs to be ruled out or established if not known already. If a peripheral schwannoma is removed in a child or adolescent, the existence of an underlying NF2 has to be ruled out actively by performing MRIs of the head and the spine. If those are negative for lesions compatible with vestibular schwannomas, meningiomas or ependymomas, schwannomatosis has to be considered in case more than one schwannoma is present.

After removal of any peripheral nerve tumour, a surveillance ultrasound screening of all extremities and brachial plexus and neck can be performed as a first diagnostic step to identify other, smaller and not palpable tumours of the peripheral nerves, which would be characteristic for an underlying phacomatosis.

#### Interdisciplinarity

We encountered a vast spectrum of localizations and clinical appearance. Therefore, this series is a collection of individual indication for surgery. In case of NF1, which is the predominant cohort where peripheral nerve surgery is necessary in the paediatric age group, interdisciplinary teams seem best prepared to manage not only the known multiple aspects of NF1 but also the possible multiple peripheral nerve tumours in all body regions. Expertise in peripheral nerve tumour surgery and function-preserving resection techniques is warranted to manage those cases well from the surgical point of view. This expertise can be provided by paediatric neurosurgeons, adult neurosurgeons and plastic surgeons and regarding manifestations in the face maxillofacial surgeons. The standard function-preserving technique for benign peripheral nerve tumours, which are the vast majority as this series, is an intracapsular tumour resection with an entry into the tumour through a part of the capsule that is not bearing any sensory or motor nerve fascicles. To achieve this, the use of nerve stimulators and higher magnification by microscope or loops is necessary.

Paediatric surgeons/general surgeons and (paediatric) orthopaedic surgeons, if they do soft tissue tumour surgery, are mostly trained according the rules of sarcoma surgery. This means wide excisions with a saved margin of tissue around the tumour and en bloc resection including the nerve bearing the tumour. While this is the correct approach for MPNST manifestations, where a loss of function is justified and agreed on with the patient and parents prior to surgery, it is not justified for a benign lesion.

On the other hand, the attempt of intracapsular resection in case of MPNST is a severe mistake which can lead to contamination of the surgical field with malignant cells and thus results in a worse prognosis for the patient. Therefore, the correct interpretation of ultrasound and MRI imaging characteristics, additional PET information and the visual aspect at surgery combined with the experience of surgeons in an interdisciplinary setting is most important to choose the correct “mode of surgery”. If in doubt, a two-stage approach with up-front biopsy or an intraoperative biopsy is necessary. However, a biopsy needs to be made according to the above-mentioned function-preserving criteria through a functionally irrelevant surface area of the tumour to avoid fascicle injury resulting in pain or motor/sensory deficits after biopsy.

As shown in this series, a significant proportion of tumours was located in the retroperitoneum or in the thoracic cavity and needed an interdisciplinary planning and approach with paediatric surgeons, including the intraoperative adherence to the criteria of sarcoma surgery as soon as malignancy is already known or confirmed by intraoperative frozen section. Neurosurgeons also need to adapt other techniques like thoracoscopic surgery to perform surgery on smaller intrathoracic tumours as minimally invasive as possible, but still applying the classical function-preserving techniques of microneurosurgery of the peripheral nerve.

**Fig. 3:**
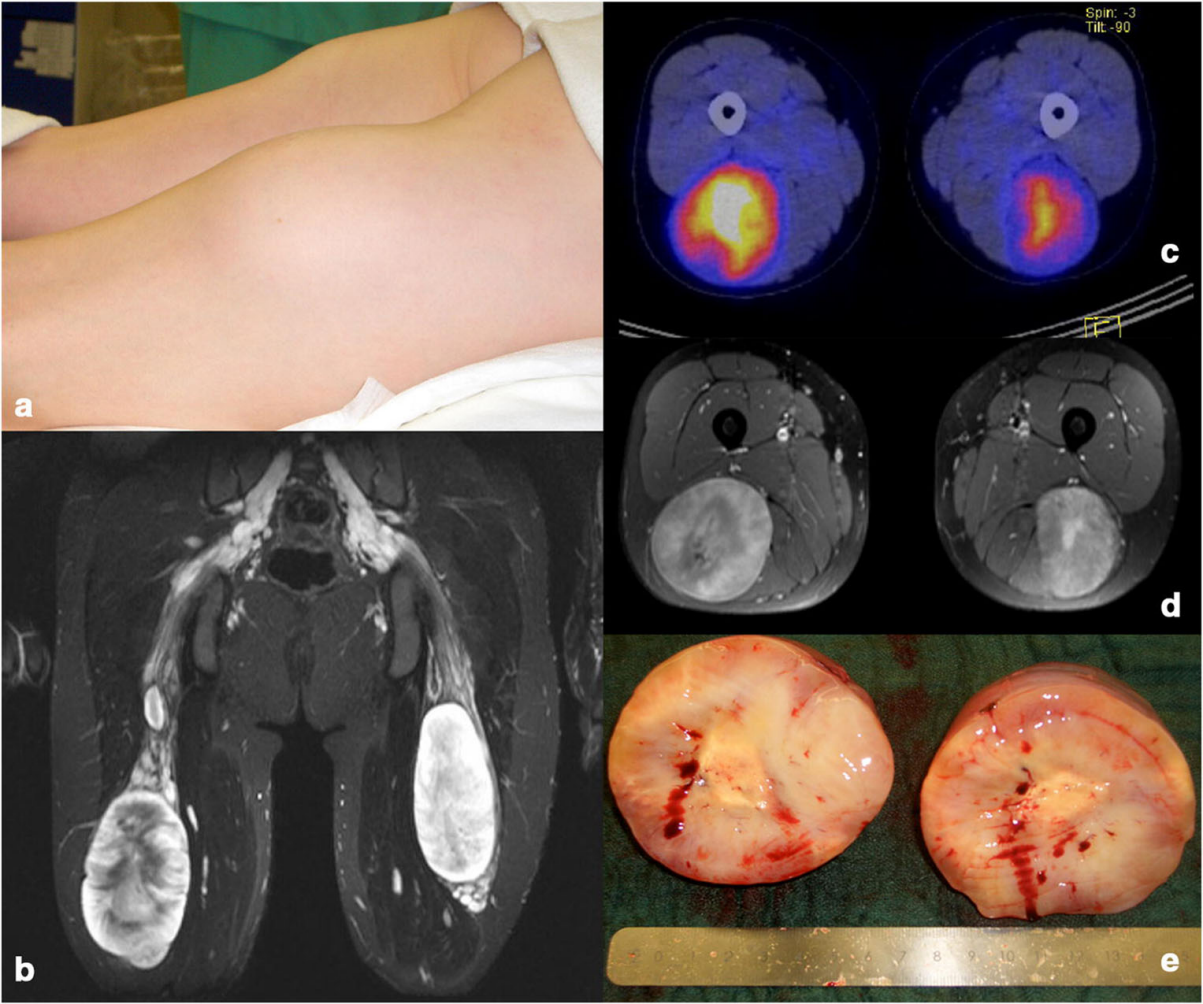
A 14-year-old girl with NF1 with a 5-year history of growing tumour masses in both sciatic nerves leading to increasing pain when walking and inability to sit normally on a chair. Tumours were judged externally to be non-resectable without loss of function. **a** Posterior aspect of both thighs in prone position at first presentation. **b** Coronal T2-weighted MRI displaying neurofibromatous transformation of the whole sciatic nerve, from which bilateral large well circumscribed tumours arise with inhomogeneous internal signal intensity. **d** Gadolinium-enhanced T1-weighted MRI shows inhomogeneous contrast uptake with central necrosis–like decrease of contrast intensity. **c** Since malignancy was suspected, a FDG PET-CT was performed showing a significantly increased glucose metabolism especially in the right tumour. **e** Right-sided tumour after microsurgical removal, cut in half. The outer tumour surface was smooth and not infiltrating the capsule, the tumour in histology rated as benign but hypercellular neurofibroma with central necrosis. There were no postoperative sensor or motor deficits and pain disappeared. The left-sided tumour was operated a few months later with the same outcome. During a follow-up of 10 years, the patient did not develop any other tumours demanding surgery anywhere in the body

**Fig. 4:**
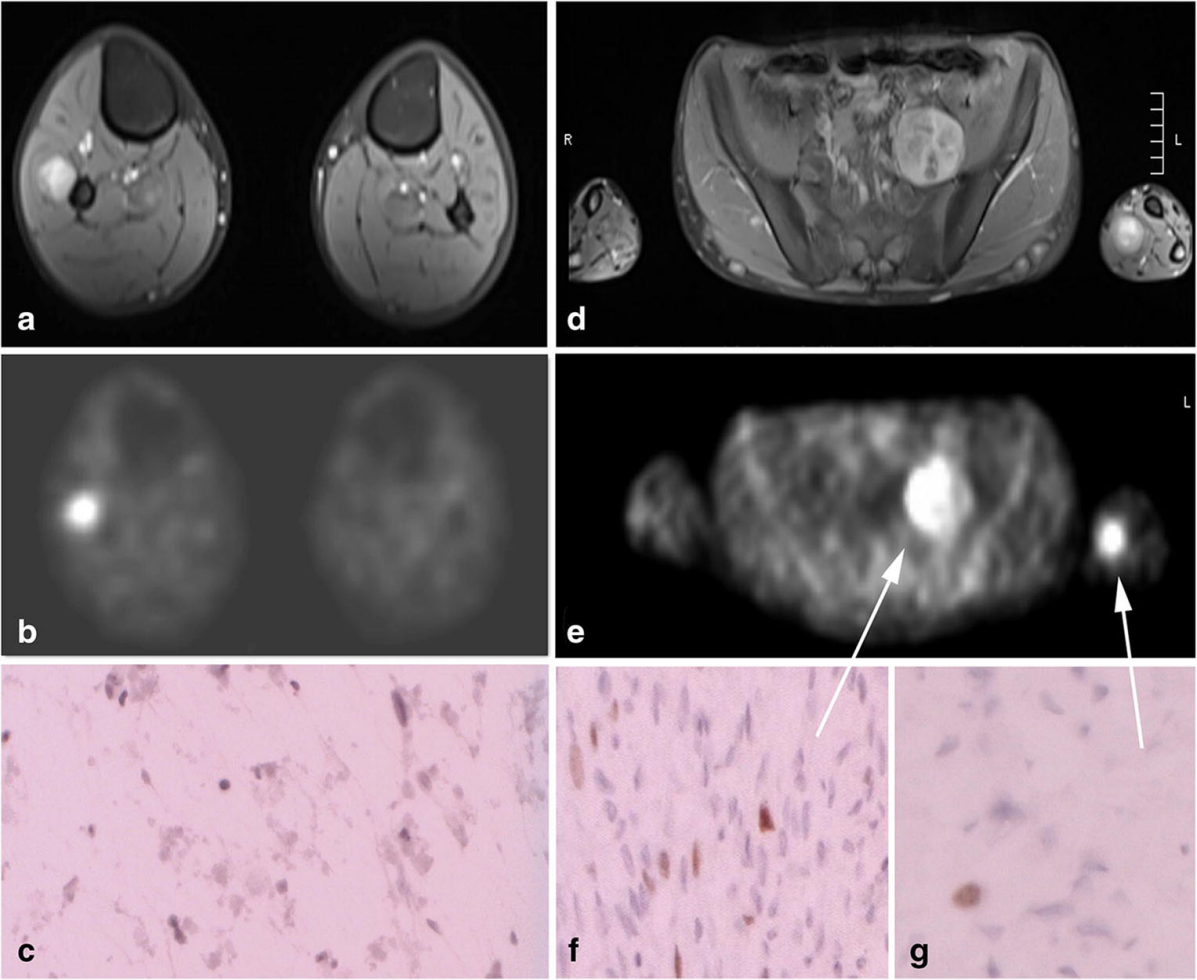
A 15-year-old adolescent boy with NF1 presenting with a painful tumour at the left forearm located at the median nerve. Screening ultrasound showed a rather high tumour load of the nerves at the extremities and a retroperitoneal left pelvic mass. **a** and **d** Whole body MRI revealed three larger tumour manifestations with inhomogeneous contrast uptake. Apart from the two mentioned above (**d**), another peroneal nerve tumour at the right lower calf 10 cm below the knee was identified (**a**). The pelvic tumour was rated suspicious for malignancy, so a PET scan was done. **b** and **e** PET scan showed high glucose metabolism in all three tumours. All tumours were removed in one surgical procedure with three separate interventions: Together with paediatric surgery, a transperitoneal approach was performed. Since frozen section from intraoperative true-cut biopsy was suspicious for malignancy, the tumour was removed according to sarcoma protocol en bloc with its capsule. Histology showed a grade 1 MPNST (**f**). The peroneal and median nerve tumours had a macroscopic appearance of benign tumours and were removed intracapsular microsurgically with preservation of function. Histology described benign neurofibromas (**c**, **g**)

The teams treating paediatric peripheral nerve tumours should furthermore include paediatric neurologists or neurologists specialised in electrophysiology in children, neurologists or radiologists specialised in peripheral nerve ultrasound and radiologist experienced in special nerve MRI (MR neurography) and PET. High-resolution imaging and MRI neurography and high-frequency ultrasound (≥ 12 MHz) are the most useful imaging techniques for the majority of lesions. If the suspicion of MPNST arises, particularly in NF1, PET options provided important preoperative information, to rule out or confirm the suspicion of malignancy and depict additional critical lesions (see Figs. 3 and 4). Proper planning seems to be the key, which also includes the clear identification of the affected nerves; the preoperative differentiation from non-affected nerves by proper clinical examination, ultrasound and electrophysiology; and in consequence, choosing the adequate approach.

### Outcome

Preoperative motor function was good in the majority of cases, and microsurgery had no significant negative impact on postoperative motor function. Furthermore, surgery was able to significantly improve pain for severely affected patients: the overall outcome was excellent with an improvement of symptoms in 75% and a deterioration in only 2.4%.

Furthermore, the non-neurological complication rate was extremely low (2 hematomas in 186 intervention, no infection). Therefore, this study provides the rationale for a surgical intervention if the indications for surgery, as mentioned above, are fulfilled. Further observation is not warranted since no strategic advantages will arise.

On the other hand, we again point out that, especially in NF1, the pure existence of an asymptomatic and non- or slowly growing neurofibroma per se is no indication for surgery, since many of those tumours behave innocently for a long time if not forever.

**Fig. 5:**
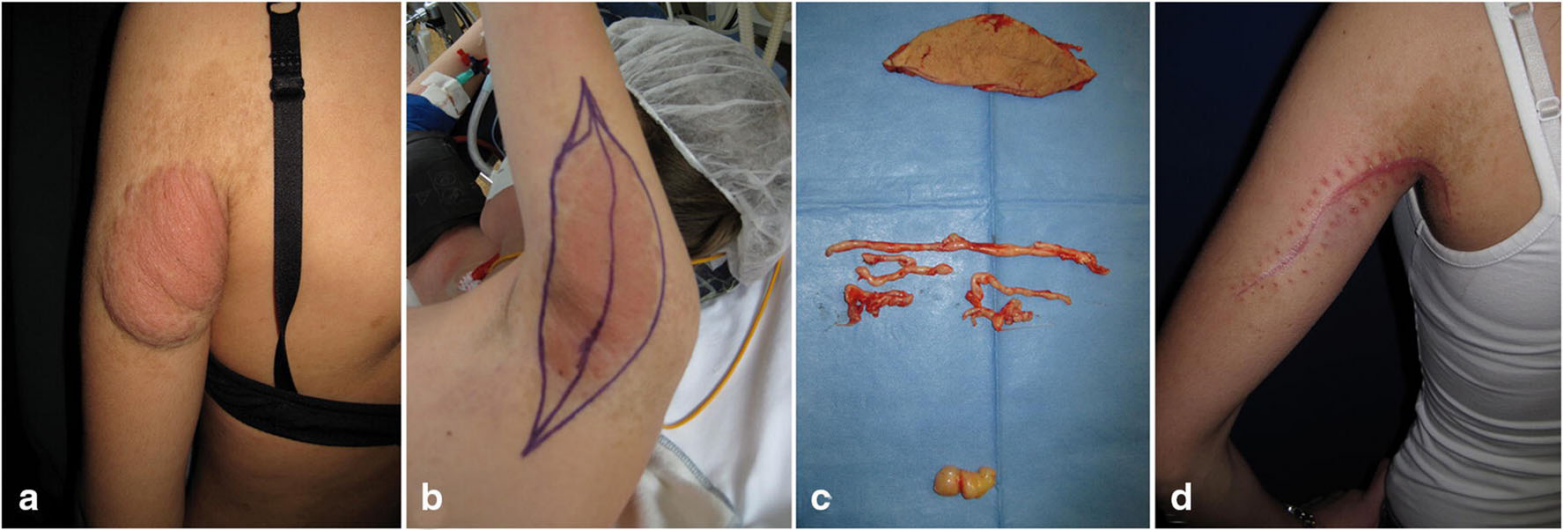
A 13-year-old girl with known NF1 bearing an ugly and meanwhile also painful cutaneous plexiform neurofibroma of the dorsal upper arm extending below the shoulder (**a**). Under the changed skin, several painful tumours were palpable in the subcutaneous tissue. **b** Intraoperative positioning and planning of skin excision. A complete excision with clean skin edges was just possible. **c** Resection result of removed skin (top), subcutaneous plexiform worm-like neurofibromas (middle) and isolated painful deep neurofibroma posterior to brachial plexus in the axilla (bottom). **d** Postoperative result 6 weeks after surgery

The only exception, apart from suspicion of malignancy, for a proactive approach due to the pure existence of a lesion, is in cutaneous PNF, as soon as they have grown enough to cause attention of parents or paediatricians. Here an early intervention that enables a complete resection with a rather small scar and easier skin closure without the need for extensive plastic skin reconstruction has a clear advantage for a small child as compared with letting the lesion grow until a large scar results or plastic reconstruction is necessary at a later time point in life. We have often experienced situations in adolescents or young adults where a complete resection has become difficult and cosmetically unsatisfying (see Fig. 5).

The most critical pathology, MPNST, still poses a diagnostic and therapeutic challenge with high morbidity and mortality [1, 19]. Since a complete resection is the only true curative option, early diagnosis is key. Compared with comparably located benign neurofibromas, MPNSTs were associated to preoperatively significant larger mean motor and sensory impairments. Since a radical surgical resection is the only curative therapeutic option, these deficits did persist or worsen. Since the vast majority of MPNST cases are associated with NF1, good clinical surveillance and patient management in close collaboration with the paediatric partners, in our setting paediatric neurologists, is of great importance. Not only from an oncologic point of view but also due to severe neurological deficits associated with the tumour and its removal, early surgical resection of suspicious lesions is of great importance. The fact that an MPNST was diagnosed only in 3 of 14 interventions performed for suspected malignancy indicates that the surveillance mechanisms established in our programme seem to work. In almost 80% of cases, we were early enough with the intervention and a malignant transformation had not yet occurred. Worrisome as it seems in this context, 3 MPNSTs (50% of the MPNST cohort) were not suspected before surgery. One patient was a primary presentation of a solitary non-NF1-associated MPNST of the median nerve, with a history of a few months and mild sensory and motor deficits. Here we proceeded with immediate surgery; however, we were not expecting a MPNST due to its rarity in non-NF1 children. The 2 other cases had NF1 and were operated on in the early period of our retrospective analysis due to larger tumours. These negative experiences prompted a high awareness of the problem in the team and a thorough application of diagnostic procedures such as regular MRI surveillance in cases with higher tumour load, PET-CT or if possible PET-MRT in case MRI showed suspicious lesions. In case of suspicion (growth plus imaging abnormalities), an early intervention is performed. Ever since then, surprises in NF1 patients have become a rarity.

Just recently, the French national guidelines have been established concerning the management of patients with NF1, advocating as well for high-resolution MRI and FDG-PET, as well as biopsy in suspected MPNST [2].

### Alternative treatment options

Radiation of benign peripheral nerve tumours is no option for peripheral nerve tumours in children, especially in the setting of an underlying phacomatosis. The alteration of a tumour suppressor gene product predisposes to secondary malignancies in the radiation field or to malignant transformation of irradiated tumour [14].

In the setting of NF1, inhibition of MAP-Kinase pathway with the so-called MEK inhibitors is a promising option of pharmacological intervention in cases a tumour reduction should be performed; however, it is surgically not possible or associated with too many risks [15, 18, 27].

## Conclusion

This retrospective large series of paediatric peripheral nerve tumour resections demonstrates that the intervention is safe and effective. Young patients benefit from early surgical resection of peripheral nerve sheath tumours that cause significant growth, pain, motor deficit or suspected malignancy. This study underlines the significance of interdisciplinary care of patients suffering from NF1, who due to the complex nature of the disease will need special requirements of surveillance, diagnostics and interdisciplinary surgery in localizations not commonly accessed by (paediatric) neurosurgeons alone.
