# Correction to: Surgical management of peripheral nerve sheath tumours in children, with special consideration of neurofibromatoses

**DOI:** 10.1007/s00381-021-05228-2

**Published:** 2021-07-19

**Authors:** Julian Zipfel, Meizer Al-Hariri, Isabel Gugel, Karin Haas-Lude, Alexander Grimm, Steven Warmann, Michael Krimmel, Victor-Felix Mautner, Marcos Tatagiba, Martin U. Schuhmann

**Affiliations:** 1 Division of Paediatric Neurosurgery, Department of Neurosurgery, University Hospital of Tübingen, Hoppe-Seyler-Str. 3, 72076 Tubingen, Germany; 2 Department of Neurosurgery, University Hospital Tübingen, Tubingen, Germany; 3 Centre for Neurofibromatosis at the Centre of Rare Diseases, University Hospital and University of Tübingen, Tubingen, Germany; 4 Department of Paediatric Neurology, University Children’s Hospital Tübingen, Tubingen, Germany; 5 Department of Neurology, University Hospital Tübingen, Tubingen, Germany; 6 Department of Paediatric Surgery, University Children’s Hospital Tübingen, Tubingen, Germany; 7 Department of Maxillofacial Surgery, University Hospital Tübingen, Tubingen, Germany; 8 Neurofibromatosis Centre Hamburg, Department of Neurology, University Medical Center Hamburg-Eppendorf, Hamburg, Germany

## Correction to: Child’s Nervous System (2020) 36:2433–2442 https://doi.org/10.1007/s00381-020-04703-6

The article *Surgical management of peripheral nerve sheath tumours in children*,* with special consideration of neurofibromatoses*, written by *Julian Zipfel*,* Meizer Al-Hariri*,* Isabel Gugel*,* Karin Haas-Lude*,* Alexander Grimm*,* Steven Warmann Michael Krimmel*,* Victor-Felix Mautner*,* Marcos Tatagiba* and* Martin U. Schuhmann*, was originally published Online First without Open Access. After publication in volume *36*, issue *10*, page *2433–2442*, the author decided to opt for Open Choice and to make the article an Open Access publication. Therefore, the copyright of the article has been changed to *© The Author(s) 2020* and the article is forthwith distributed under the terms of the Creative Commons Attribution 4.0 International License, which permits use, sharing, adaptation, distribution and reproduction in any medium or format, as long as you give appropriate credit to the original author(s) and the source, provide a link to the Creative Commons licence and indicate if changes were made. The images or other third-party material in this article are included in the article’s Creative Commons licence, unless indicated otherwise in a credit line to the material. If material is not included in the article’s Creative Commons licence and your intended use is not permitted by statutory regulation or exceeds the permitted use, you will need to obtain permission directly from the copyright holder. To view a copy of this licence, visit http://creativecommons.org/licenses/by/4.0.

